# Multi-omics analysis of *Bacillus*-associated suppression of *Bursaphelenchus xylophilus* disease symptoms and candidate defense responses in *Pinus thunbergii*

**DOI:** 10.3389/fpls.2026.1854108

**Published:** 2026-10-14

**Authors:** Yue Zhang, Kexiang Zhi, Zhiyuan Chen, Jing Han, Bin Liu, Wei Ma, Zhihua Liu, Ling Ma, Yibo An

**Affiliations:** 1 Department of Forest Protection, College of Forestry, Northeast Forestry University, Harbin, China; 2 College of Life Sciences, Northeast Forestry University, Harbin, China; 3 Chongqing Forestry Investment Development Co. Ltd., Chongqing, China; 4 National Reserve Forest Research Institute, Chongqing, China; 5 College of Land and Environment, Shenyang Agricultural University, Shenyang, China; 6 College of Pharmacy, Heilongjiang University of Chinese Medicine, Harbin, China

**Keywords:** pine wilt disease, *Bursaphelenchus xylophilus*, Pinus thunbergii, biocontrol, *Bacillus velezensis*, multi-omics analysis

## Abstract

**Introduction:**

Pine wilt disease (PWD), caused by *Bursaphelenchus xylophilus*, is a devastating threat to pine forests worldwide. This study evaluated the biocontrol potential of two Bacillus strains, *Bacillus thuringiensis* B122 and *Bacillus velezensis* B223, isolated from post-logging rhizosphere soil, to suppress PWD symptoms and characterize associated host responses in Pinus thunbergii.

**Methods:**

Root drenching with bacterial suspensions was applied, followed by nematode inoculation. Disease severity and relative control efficacy were assessed. Early needle assays measured hydrogen peroxide, malondialdehyde, peroxidase, and superoxide dismutase responses. Integrated transcriptomic and metabolomic analyses were used to identify candidate molecular associations underlying differential efficacy.

**Results:**

Root drenching with bacterial suspensions significantly reduced disease severity after nematode inoculation. B223 exhibited superior relative control efficacy (76.1%) compared with B122 (40.8%). Early needle assays revealed strain-associated differences in hydrogen peroxide, malondialdehyde, peroxidase, and superoxide dismutase responses. Integrated transcriptomic and metabolomic analyses identified candidate molecular associations underlying this differential efficacy. Both strains were associated with enrichment of defense-related pathways, including MAPK signaling, phenylpropanoid biosynthesis, and flavonoid biosynthesis. B223 showed distinct enrichment of the plant hormone signal transduction pathway, including jasmonate/ethylene (JA/ET)-related annotations, and broader metabolic reprogramming.

**Discussion:**

We propose a cautious working model in which B223-associated symptom suppression may involve coordinated hormone-related transcriptional responses, MAPK signaling, and downstream phenylpropanoid/flavonoid metabolism. Collectively, these data support B223 as a promising biocontrol candidate against PWD.

## 1 Introduction

Pine wilt disease (PWD) is caused by the pinewood nematode *Bursaphelenchus xylophilus*, which is transmitted to susceptible pines through feeding wounds made by *Monochamus* spp. adults. After entry, nematodes migrate through cortical and xylem resin canals, feed and reproduce within stem tissues, and are associated with progressive cavitation and embolism of water-conducting tracheids. Loss of xylem hydraulic function precedes needle discoloration, wilting, and, in susceptible trees, rapid death (Hao et al., 2023; Wang et al., 2022b). Current management primarily relies on quarantine, removal and incineration of infested trees, trunk injection of nematicides, and vector control (Wang et al., 2022a; Zhang et al., 2022b). These measures are often costly, labor-intensive, environmentally damaging, and carry the risk of driving nematode resistance, failing to provide a fundamental solution for containment (Pires et al., 2022). These limitations hinder effective and sustainable containment of the disease (Futai, 2013). Recent studies suggest that enhancing tree innate resistance represents a promising and sustainable alternative for disease management. For instance, *Bacillus velezensis* strain BY6 has been shown not only to promote poplar growth but also to significantly improve its resistance against pathogens (Zhang et al., 2022a). Similarly, certain pine endophytic bacteria can induce systemic resistance and reduce PWD severity(D. Li et al., 2023). Collectively, these findings highlight the potential of beneficial microorganisms in activating the host’s endogenous defense system, offering a viable and eco-friendly strategy to increase pine resistance to PWD.

Advancements in high-throughput sequencing have drawn increasing attention to the roles of pine rhizosphere and endophytic microbial communities in both the development and suppression of PWD (Wang et al., 2019). Notably, certain bacterial groups have been shown to correlate significantly with pine disease resistance(Xue et al., 2019). Among these, *Bacillus* spp. represent one of the most extensively studied and widely applied genera for plant growth promotion and biocontrol (Tang et al., 2024). Their ability to form endospores confers strong environmental adaptability, and they are amenable to formulation and field application (Bellamy et al., 2022). Numerous studies have demonstrated that *Bacillus* can suppress pathogens through the secretion of diverse metabolites such as lipopeptides, proteases, polysaccharides, and volatile organic compounds (Hazarika et al., 2019; Ye et al., 2023). Meanwhile, they induce systemic resistance and cross-protection in plants by activating signaling pathways like salicylic acid and jasmonic acid, achieving favorable results in controlling multiple crop diseases (Gogoi et al., 2025; Maalik et al., 2023). For example, pine seedlings treated with *B. subtilis* JCK-1398 exhibited a significantly reduced disease index under nematode challenge. This was accompanied by enhanced accumulation of defense-related secondary metabolites such as the lignan pinoresinol, upregulation of defense genes, and activation of signaling pathways including mitogen-activated protein kinase (MAPK) (Mannaa et al., 2024). Furthermore, this strain indirectly remodeled the rhizosphere microbiome and nematode-associated microbial community by altering root exudates, collectively improving overall pine health (Mannaa et al., 2025). An engineered derivative of the pine endophyte *Bacillus toyonensis* Bxy19 was designed to express a Cry nematicidal protein and reduced seedling mortality under greenhouse conditions. This engineered strain demonstrated the ability to colonize and migrate within pine needles and xylem over extended periods, highlighting the potential of engineered *Bacillus* strains for PWD management (Li et al., 2023). Moreover, recent studies have identified *B. velezensis* strains isolated from endophytic communities of *Pinus tabuliformis* and other pines that show notable nematocidal and reproduction-suppressing activities *in vitro* (Asaturova et al., 2022). These findings provide promising new resources for developing high-efficacy *Bacillus*-based biocontrol agents against PWD.

Based on previous evidence that beneficial rhizosphere bacteria can suppress pine wilt disease symptoms and modulate host defense responses, we tested the following hypotheses: (1) Rhizosphere-derived *Bacillus* strains differ in their ability to suppress pine wilt disease symptoms in *P. thunbergii* following inoculation with *B. xylophilus*; (2) Strains with higher biocontrol efficacy are associated with more coordinated host defense responses, including antioxidant regulation, defense-related enzyme activation, and immune signaling; (3) And the superior protective effect of an effective strain is associated with distinct transcriptional and metabolic reprogramming of defense-related pathways, particularly hormone signaling, phenylpropanoid biosynthesis, and flavonoid metabolism. To test these hypotheses, two rhizosphere-derived strains, *B. thuringiensis* B122 and *B. velezensis* B223, were evaluated using disease assays, physiological measurements, transcriptomic profiling, and metabolomic analysis. This integrated approach was used to identify candidate microbial resources and generate testable mechanistic hypotheses for developing *Bacillus*-based biocontrol strategies against PWD.

## 2 Materials and methods

### 2.1 Bacterial isolation, cultivation, and activity assay

The bacterial strains used in this study were isolated from rhizosphere soil samples collected from a pine forest in Xingqiao Town, Liangping District, Chongqing Municipality, China. For isolation, 10 g of soil was suspended in 100 mL of sterile water and heated at 90°C for 2 hours to enrich for spore-forming bacteria. The suspension was then serially diluted (10- and 50-fold), and 200 µL aliquots were spread onto Luria-Bertani (LB) plates. Following incubation at 28°C for 3 days, distinct single colonies were selected and purified by repeated streaking on fresh LB plates. For molecular identification, genomic DNA was extracted from pure cultures using a Bacterial DNA Kit (D3350, Omega, USA). The 16S rRNA gene was amplified via PCR using the universal bacterial primers 799F (5′-AACMGGATTAGAGATCKG-3′) and 1193R (5′-ACGTCATCCCCACCTTCC-3′). The resulting PCR products were purified and sequenced by Sangon Biotech (Shanghai, China). The obtained sequences were compared against reference databases using the BLAST online tool and the Ribosomal Database Project. A phylogenetic tree was constructed to confirm taxonomic affiliations. The strains were deposited at the Guangdong Microbial Culture Collection Center (GDMCC) with accession numbers GDMCC No. 66033 and 66034.

### 2.2 Nematode culture and harvest

The *B. xylophilus* strain Nxy61, preserved in the laboratory of the Chinese Academy of Forestry, was used in this study. Nematodes were cultured and propagated on potato dextrose agar (PDA) plates pre-inoculated with the fungus *Botrytis cinerea*. After the nematodes had consumed the fungal mycelia, approximately 15 mL of 1% PBST (phosphate-buffered saline containing 0.05% Tween-20) was added to each plate. The nematodes were dislodged by gentle pipetting, and the suspension was collected into a 15 mL centrifuge tube. This washing step was repeated twice. The combined nematode suspension was filtered through a 100 μm mesh to remove residual fungal hyphae. The filtrate was then centrifuged at 5,000 rpm for 5 minutes. The pellet was washed three times with sterile water. Nematodes were counted under a microscope and subsequently resuspended in sterile water to a final working concentration of 100 nematodes per µL.

### 2.3 Plant inoculation experiment

The selected endophytic bacterial strains were cultured in Luria–Bertani broth at 28 °C with shaking at 180 rpm for 48 hours. Following incubation, the bacterial suspensions were serially diluted (10-fold, 100-fold, and 1000-fold). A 10 µL aliquot from each dilution was loaded into a hemocytometer, and approximate cell density was determined using a Zeiss Axio Observer microscope with a 40× objective and 10× eyepieces, giving a total magnification of 400×. Based on the cell counts, the suspension concentrations were precisely adjusted to 1 × 10^7^ CFU/mL via gradient dilution. Three-year-old *P. thunbergii* seedlings, sourced from Junan County, Linyi City, Shandong Province, were used. Each seedling was planted in a plastic pot (22 cm × 20 cm × 19 cm) containing approximately 5 L of nutrient soil. The substrate mixture of straw, vermiculite, and perlite (2:3:5, v/v/v) was autoclaved at 121 °C for 20 min prior to use. All plants were maintained in a greenhouse at the Chinese Academy of Forestry under controlled conditions (25 °C, 70% relative humidity, natural light). Seedlings of uniform growth vigor were selected and divided into treatment and control groups, with six biological replicates per group. Each pot in the treatment group received 100 mL of the bacterial suspension (1 × 10^7^ CFU/mL), while control pots received an equal volume of sterile water. The plants were maintained under the aforementioned greenhouse conditions. Seven days after bacterial treatment, a 3 mm diameter hole was drilled into the stem of each seedling at a height of 15 cm above the soil surface, taking care not to penetrate the stem completely. Using a micropipette, 100 µL of nematode suspension (containing approximately 10,000 individuals) was injected into the hole, which was subsequently sealed with parafilm.

### 2.4 Disease index and control efficacy assessment

Disease severity was evaluated 60 days after nematode inoculation. Symptoms on *P. thunbergii* seedlings were classified into six grades (0–5) based on the percentage of wilted foliage: Grade 0: No visible symptoms; Grade 1: <10% of needles wilted; Grade 2: 10–30% wilted; Grade 3: 30–50% wilted; Grade 4: >50% wilted; Grade 5: Plant dead. The disease index (DI) and relative control efficacy (RCE) were calculated according to the following formulas:


$$\text{DI}=100\times \sum \frac{\left(\text{Number of plants in each grade}\times \text{Grade value}\right)}{\left(\text{Total number of plants surveyed}\times \text{Highest grade value}\right)}$$

$$\text{RCE}(\%)=\frac{\left({\text{DI}}_{\text{control}}-{\text{DI}}_{\text{treatment}}\right)}{{\text{DI}}_{\text{control}}}\times 100$$

### 2.5 Assessment of physiological changes in pine seedlings following bacterial treatment

To evaluate the temporal physiological response of *P. thunbergii* to bacterial treatment, needle samples were randomly collected from seedlings at 1, 3, 5, and 7 days post-inoculation with the bacterial suspension. Sampling was conducted carefully to avoid affecting subsequent plant growth. For each time point, collected needles from each treatment group were pooled, thoroughly mixed, and divided into three biological replicates. All samples were immediately flash-frozen in liquid nitrogen and stored at –80 °C until analysis. Frozen tissue was used to quantify key physiological and defense-related parameters, including hydrogen peroxide (H_2_O_2_) content, malondialdehyde (MDA) level, and the activities of peroxidase (POD) and superoxide dismutase (SOD). Measurements were performed using commercially available assay kits according to the manufacturers’ instructions. These analyses aimed to elucidate the impact of bacterial treatment on the host plant’s oxidative status and early defense activation.

### 2.6 Transcriptomic analysis of pine seedlings following bacterial treatment

Total RNA was extracted from frozen needle samples using TRIzol reagent (Invitrogen, USA). RNA concentration and purity were assessed with a Nanodrop 2000 spectrophotometer (Thermo Fisher Scientific). RNA-seq library preparation was performed using the Illumina^®^ Stranded mRNA Prep, Ligation kit (San Diego, USA). Sequencing was carried out on a NovaSeq X Plus platform (Illumina) in a 150-bp paired-end mode using the NovaSeq Reagent Kit. All library preparation and sequencing steps were conducted by Shanghai Majorbio Biopharm Biotechnology Co., Ltd. (Shanghai, China), following the manufacturer’s protocols. The obtained clean reads were aligned to the *P. thunbergii* reference genome using HISAT2 v2.2.1 with the --sensitive --no-discordant --no-mixed preset. Read counts per gene were quantified using featureCounts v2.0.1 with default parameters. Genes with a total read count below 10 across all samples were excluded from downstream analysis. Differential expression analysis was performed using the DESeq2 package (v1.34.0) in R (v4.1.2), with genes considered differentially expressed if they met the thresholds of |log2(fold change)| ≥ 1 and an adjusted *p*-value (FDR, Benjamini-Hochberg correction) < 0.05. Gene Ontology (GO) and Kyoto Encyclopedia of Genes and Genomes (KEGG) pathway enrichment analyses of the differentially expressed genes were conducted using the online Majorbio Cloud Platform (https://cloud.majorbio.com/).All subsequent read lengths are stored in the Short Read Archive (SRA) database (http://www.ncbi.nlm.nih.gov/sra/), accessible via accession numbers PRJNA1429861.

### 2.7 RT-qPCR

To validate the transcriptome sequencing results, nine target genes were randomly selected for mRNA real-time quantitative PCR (RT-qPCR) analysis. Total RNA was extracted using RNAiso-Plus reagent (Takara), and cDNA was synthesized with the EasyScript One-Step gDNA Removal and cDNA Synthesis SuperMix (TransGen) after genomic DNA removal. The qPCR reactions were performed using AceQ qPCR SYBR Green MasterMix (Vazyme) on a Bio-Rad CFX96 system. All primer sequences are listed in **Supplementary Table 1** and were synthesized by Sangon Biotech. *U2af* was used as the internal reference gene for mRNA, and three biological replicates were conducted for each sample. Relative expression levels were calculated using the 2^−ΔΔCt^ method (Livak and Schmittgen, 2001).

### 2.8 Metabolomic analysis of *P. thunbergii* following *B. xylophilus* inoculation

Following the procedures described in Section 2.3, *P. thunbergii* seedlings were treated with bacterial suspensions and subsequently inoculated with *B. xylophilus*. Sixty days after nematode inoculation, stem segments were collected from regions located 3 cm above and 3 cm below the inoculation point for metabolomic profiling. Metabolites were extracted from the samples using a methanol-water solution (4:1, v/v). The extracts were then analyzed by liquid chromatography-tandem mass spectrometry (LC-MS/MS) on a Thermo Fisher UHPLC-Orbitrap Exploris 240 system, operating in both positive and negative ionization modes. Raw data were processed with Progenesis QI software (Waters Corporation, Milford, USA) for feature detection and alignment. Multivariate statistical analysis, including principal component analysis (PCA) and partial least squares-discriminant analysis (PLS-DA), was performed using the ropls package (version 1.6.2) in R, with 7-cycle cross-validation used to evaluate model stability. Model quality was reported using R2X, R2Y, Q2, and their cumulative values when available. Differential metabolites were screened based on a variable importance in projection (VIP) score ≥ 1 and a p-value < 0.05. Metabolite features were annotated by matching accurate mass, MS/MS fragmentation spectra, and retention information against public and in-house databases. Annotation confidence was assigned according to the Metabolomics Standards Initiative, MSI. Metabolites confirmed by authentic standards were classified as MSI level 1; metabolites annotated by accurate mass and MS/MS spectral similarity were classified as MSI level 2; putatively characterized compound classes were classified as MSI level 3. Only metabolites with reliable annotation levels were used for pathway enrichment and biological interpretation. Subsequently, KEGG pathway enrichment analysis was performed to identify biologically relevant metabolic pathways. All subsequent read lengths are stored in the Short Read Archive (SRA) database (http://www.ncbi.nlm.nih.gov/sra/), accessible via accession numbers PRJNA1429863.

### 2.9 Statistical analysis

All quantitative data are presented as mean ± standard deviation (SD). Statistical analyses were performed using GraphPad Prism 7. Differences between two groups were assessed by unpaired, two-tailed Student’s *t*-tests. For physiological outcomes, groups within each strain (control and 1, 3, 5, and 7 d) should be assessed for normality using the Shapiro–Wilk test and for homogeneity of variances using Levene’s test before one-way analysis of variance. A probability *p*-value of less than 0.05 was considered statistically significant. In graphical representations, significant differences between groups are denoted by asterisks (*p* < 0.05) or distinct lowercase letters.

## 3 Results

### 3.1 Screening of strains B122 and B223

Previous studies have shown that high-intensity and low-intensity logging alter soil bacterial and fungal communities, significantly enhancing the accumulation of total nitrogen and available nitrogen in the soil (Li, 2024; Li et al., 2024). Building on this, we isolated and identified bacteria from soil samples collected across three plot types. A total of 371 bacterial strains were obtained, belonging to four species: *Bacillus velezensis*, *Bacillus amyloliquefaciens*, *Bacillus thuringiensis*, and *Bacillus subtilis*, with *B. velezensis* being the most abundant (**Supplementary Table 2**). The isolated strains were assessed for multiple functional traits, including nitrogen fixation capacity, phosphate solubilization, hemolytic activity, indole-3-acetic acid (IAA) production, and motility. Strains exhibiting distinct differences in these traits were selected for further analysis. The results indicated that strain B223 consistently outperformed strain B122 across all measured parameters (**Figure 1**; **Supplementary Figures S1**, **S2**).

**Figure 1:**
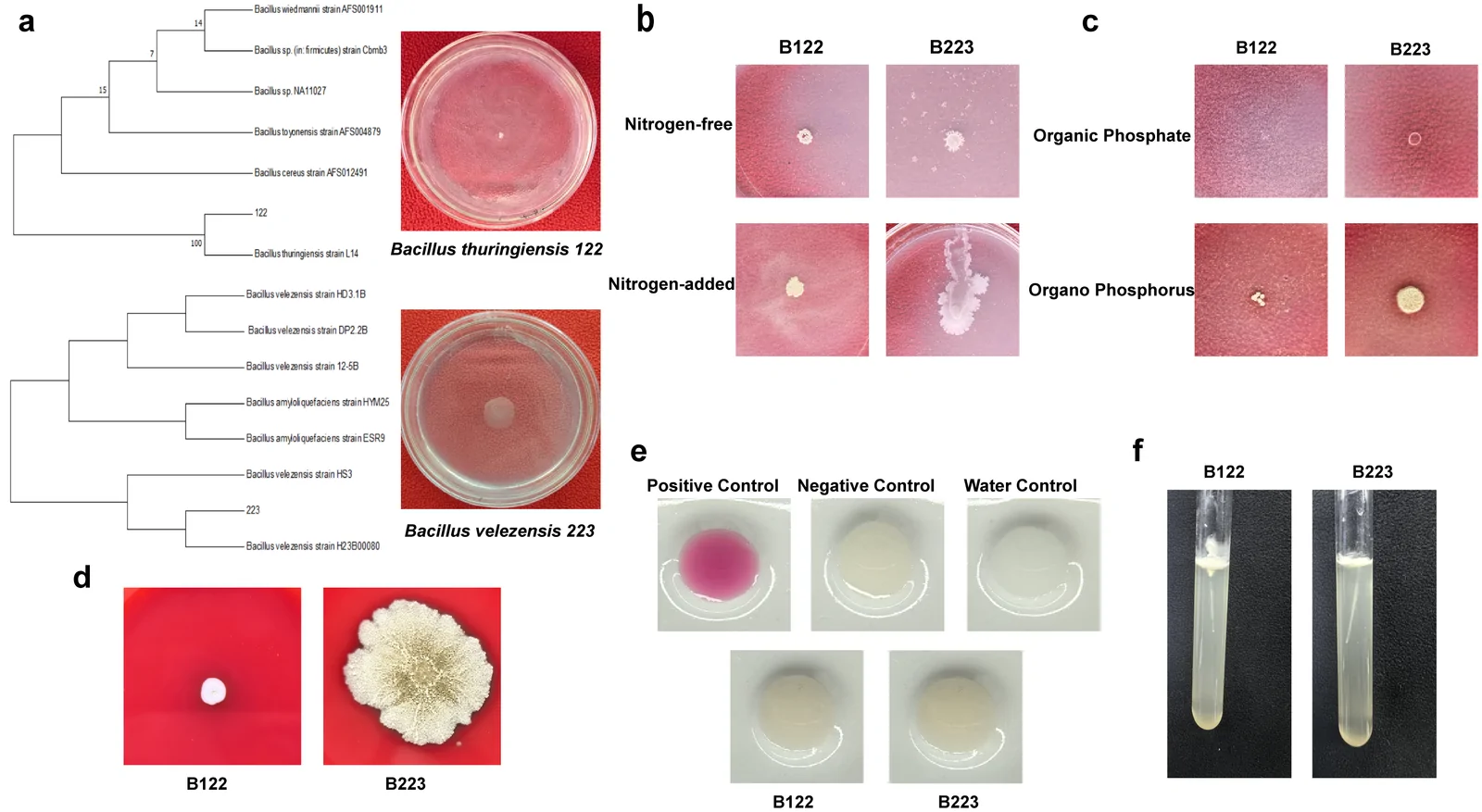
Identification of B122 and B223 and determination of biocontrol capacity. **(A)** Phylogenetic tree and morphological identification. **(B)** Nitrogen-fixing capacity. **(C)** Phosphorus-solubilizing ability. **(D)** Hemolytic activity. **(E)** Indole-3-acetic acid (IAA) production capacity. **(F)** Motility.

### 3.2 Evaluation of disease resistance in *P. thunbergii* induced by strains B122 and B223

After 48 h cultivation in Luria–Bertani broth, B122 and B223 cell suspensions were applied by root drenching. After a seven-day co-cultivation period to allow bacterial establishment, *B. xylophilus* were inoculated into the stems via a pin-puncture method. Disease progression was monitored for 60 days post-inoculation. As shown in **Figure 2**, control seedlings displayed pronounced wilting symptoms. In contrast, seedlings treated with either B122 or B223 exhibited markedly reduced wilting severity. Quantitative analysis based on the disease index revealed a score of 71 for the control group. Treatment with B122 and B223 significantly lowered the disease indices to 42 and 17, respectively, corresponding to relative control efficacies of 40.8% and 76.1% (**Table 1**). These results demonstrate that root application of B122 and B223 suspensions is associated with measurable suppression of pine wilt disease symptoms in *P. thunbergii.* Because direct nematicidal activity was not assessed in this experiment, the observed protection cannot yet be attributed exclusively to host-mediated resistance.

**Figure 2:**
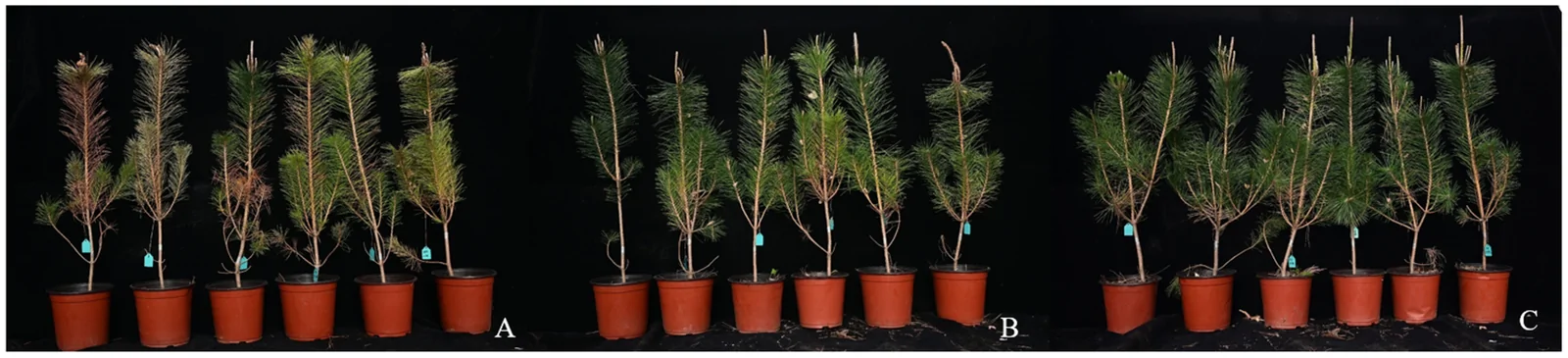
Wilting degree of *P. thunbergii* under B122 and B223 treatment with advance. **(A)** The wilt severity of *P. thunbergii* with pine wood nematode alone. **(B, C)** The *P. thunbergii* inoculated with *B. xylophilus* 7 days after root irrigation treatment with strain B122 and B223.

**Table 1:** Control results of pine wilt disease using bacterial B122 and B223.

Experimental treatments	Disease indices	Relative control efficacies
B122 + Nxy61	42	40.8%
B223 + Nxy61	17	76.1%
Nxy61	71	

### 3.3 Analysis of defense-related enzyme activities in *P. thunbergii* in response to B122 and B233

To assess the physiological impact of bacterial treatments on host plants, we measured the activities of key defense-related enzymes in *P. thunbergii* seedlings at 1, 3, 5, and 7 days post-treatment with bacterial suspensions. Distinct response patterns were observed between the two strains (**Figure 3**). Treatment with B122 induced a significant increase in H_2_O_2_ content. In contrast, H_2_O_2_ levels in B223-treated plants remained largely unchanged or showed a slight decrease. Both treatments led to a significant rise in malondialdehyde (MDA) content. Interestingly, the activities of peroxidase (POD) superoxide dismutase (SOD) followed a different trend, with both enzymes exhibiting significantly lower activities in treated plants compared to the control. In the B223 group, POD activity showed an initial increase before gradually declining. In the B122 group, SOD activity increased progressively over time, though it remained below control levels. These results indicate that B122 and B223 induce distinct redox- and defense-enzyme response patterns. However, the present measurements do not establish whether the B122-associated H_2_O_2_ accumulation represents beneficial defense signaling, excessive oxidative stress, or both.

**Figure 3:**
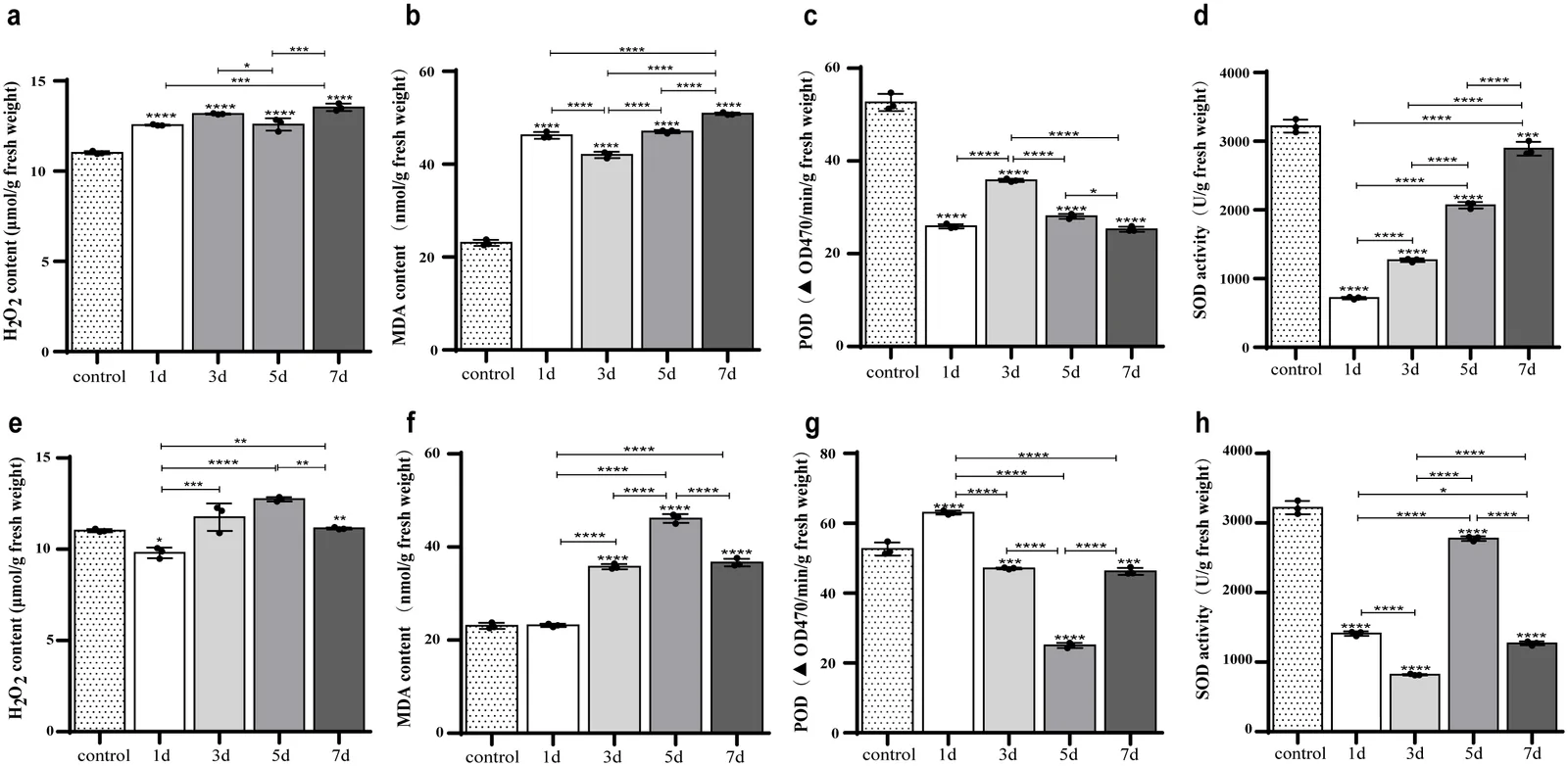
Physiological responses of *P. thunbergii* to treatments with B122 and B223. **(a)** H_₂_O_₂_ content under B122 treatment. **(b)** Malondialdehyde (MDA) content under B122 treatment. **(c)** Peroxidase (POD) activity under B122 treatment. **(d)** Superoxide dismutase (SOD) activity under B122 treatment. **(e)** H_₂_O_₂_ content under B223 treatment. **(f)** Malondialdehyde (MDA) content under B223 treatment. **(g)** Peroxidase (POD) activity under B223 treatment. **(h)** Superoxide dismutase (SOD) activity under B223 treatment. Error bars represent standard deviation (SD) of six biological replicates. Statistical significance was determined using one-way ANOVA followed by Tukey's HSD post-hoc test. Error bars represent standard deviation (SD); *, *p* < 0.05; **, *p* < 0.001; ***, ****, *p* < 0.0001, as determined by Student's t-test.

### 3.4 Transcriptomic analysis of *P. thunbergii* in response to B122 and B233

RNA sequencing generated 27 needle libraries (**Supplementary Table 3**). Principal component analysis separated the treatment/time groups and demonstrated good clustering among most biological replicates (**Figure 4A**). Across the comparisons with the control, B122 and B223 were associated with 4,669 and 5,166 differentially expressed genes, respectively, with detailed numbers of up- and down-regulated genes provided in **Figures 4B, C** and **Supplementary Figure 3**. Rather than focusing solely on the number of differentially expressed genes, we identified convergent transcriptional responses between the two treatments, particularly in defense-related signaling and secondary metabolism pathways (**Figure 4D**). To further validate the transcriptomic results, nine representative differentially expressed genes were randomly selected for RT-qPCR analysis. The expression patterns obtained by RT-qPCR were highly consistent with the RNA-sequencing data, supporting the reliability of the transcriptomic analysis (**Supplementary Figure 4**).

**Figure 4:**
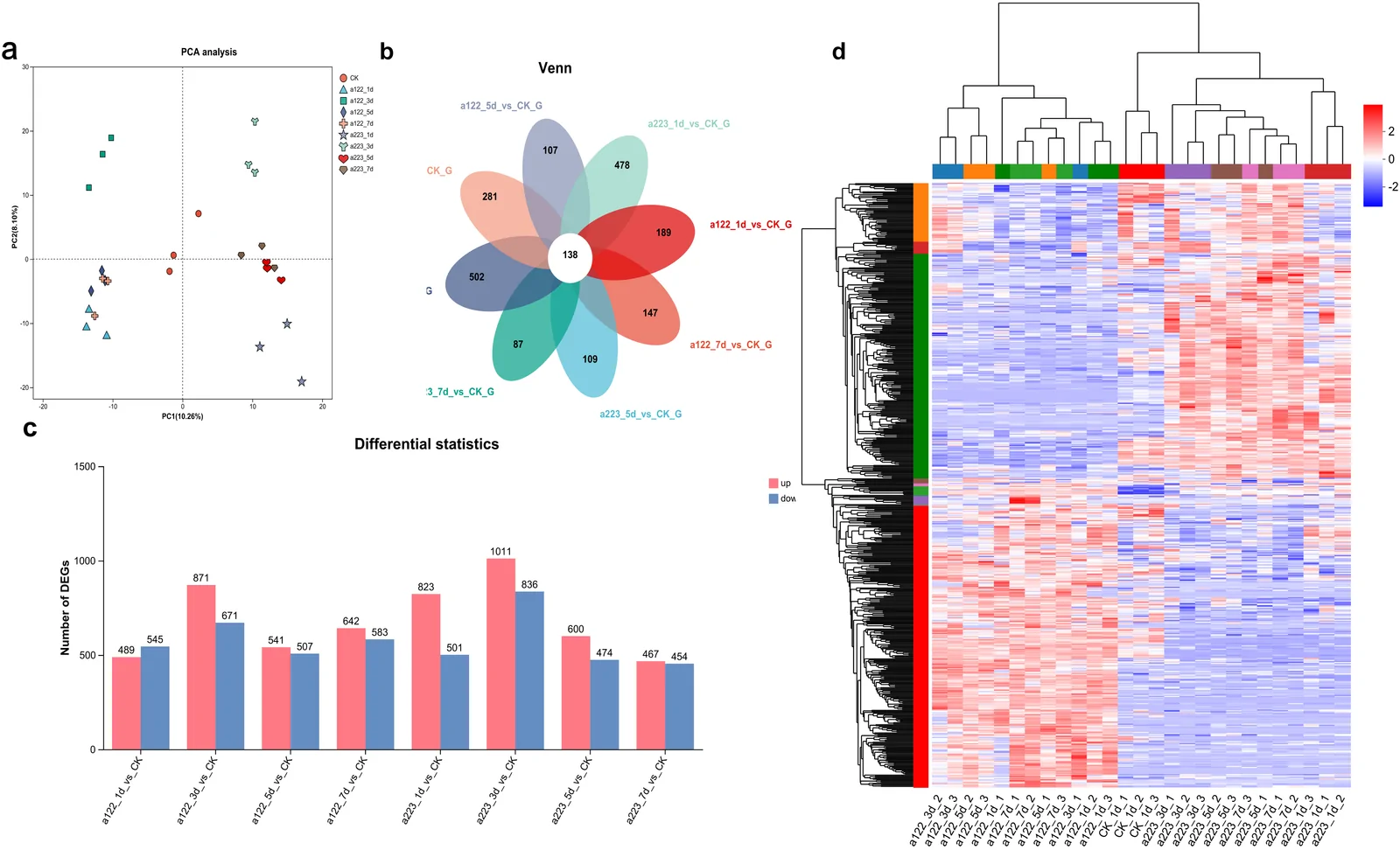
Transcriptomic analysis of *P. thunbergii* responses to strains B122 and B223. **(A)** Principal component analysis (PCA). **(B)** Venn diagram of DEGs across time points. **(C)** Counts of DEGs across time points. **(D)** Heatmap of DEGs.

Functional enrichment analysis revealed biologically relevant response modules. Both B122 and B223 treatments showed enrichment of protein kinase/phosphorylation-related terms, mitogen-activated protein kinase (MAPK) signaling, phenylpropanoid biosynthesis, and flavonoid biosynthesis pathways (**Figure 5**). In addition, B122 showed enrichment in plant–pathogen interaction and glutathione metabolism, whereas B223 exhibited distinct enrichment in nitrogen metabolism and plant hormone signal transduction (**Supplementary Figure 3**). These results indicate candidate transcriptional response networks associated with bacterial treatments.

**Figure 5:**
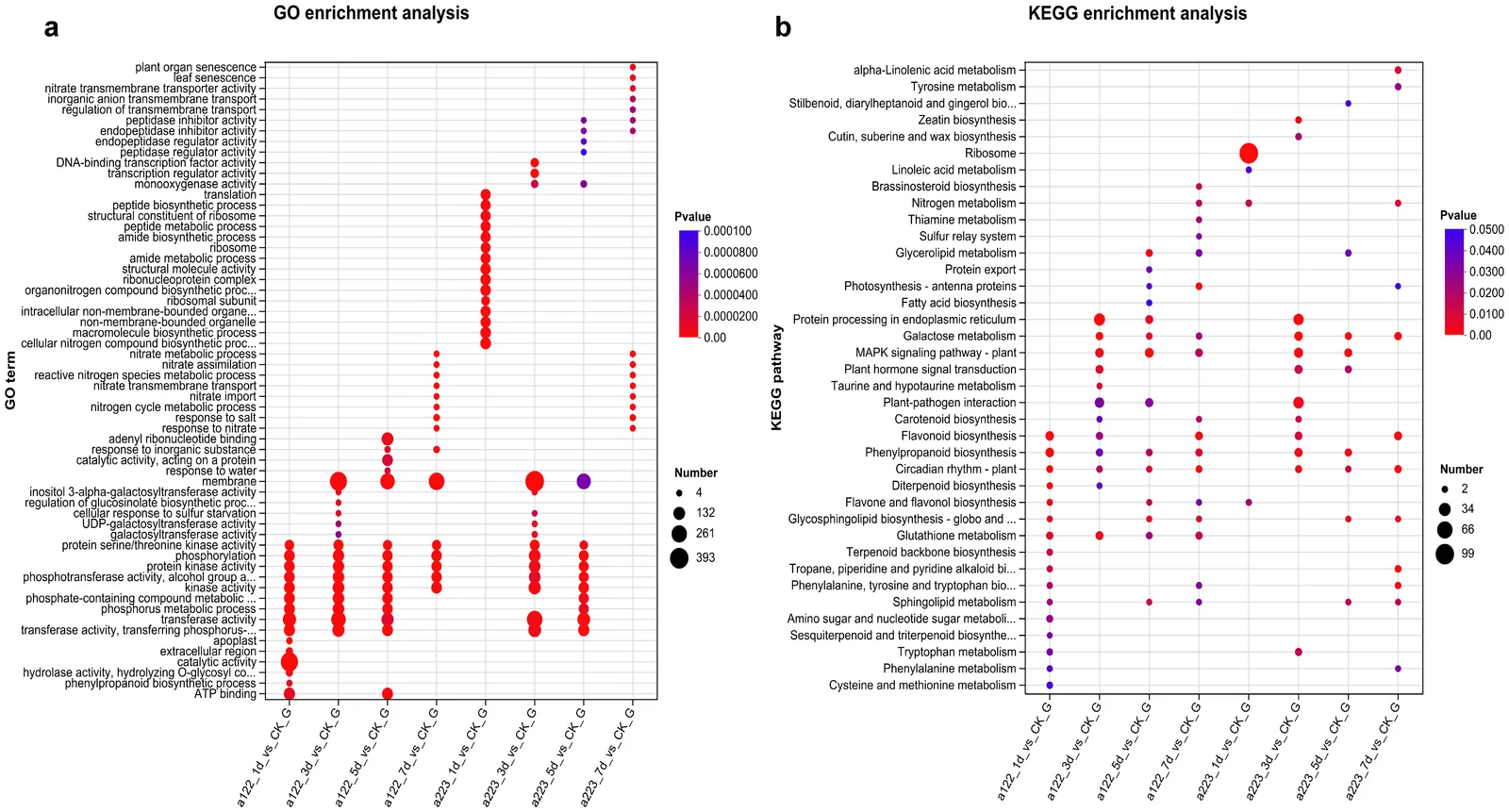
Top 15 enriched GO and KEGG analyses of DEGs. **(A)** GO analysis. **(B)** KEGG analysis.

### 3.5 Effect of B122 and B223 treatment on the metabolome of *P. thunbergii.*

Stem metabolomics at 60 d showed broad treatment-associated differences after nematode inoculation. Principal-component and partial least squares discriminant score plots separated treated samples from the control, but the available two-component supervised model had R²X(cumulative) = 0.262, R²Y(cumulative) = 0.268, and Q²(cumulative) = 0.161, indicating limited predictive ability; it is therefore used only as exploratory visualization (**Supplementary Figure 5**). The most biologically relevant observation was that B223 was associated with a larger set of differential metabolite features than B122 in both upper and lower stem segments. Lipids, phenylpropanoids/polyketides, organic oxygen compounds, organic acids, organoheterocyclic compounds, and benzenoids predominated among annotated features. These are feature-level associations, and compound identities and biological activities require targeted confirmation (**Figure 6**).

**Figure 6:**
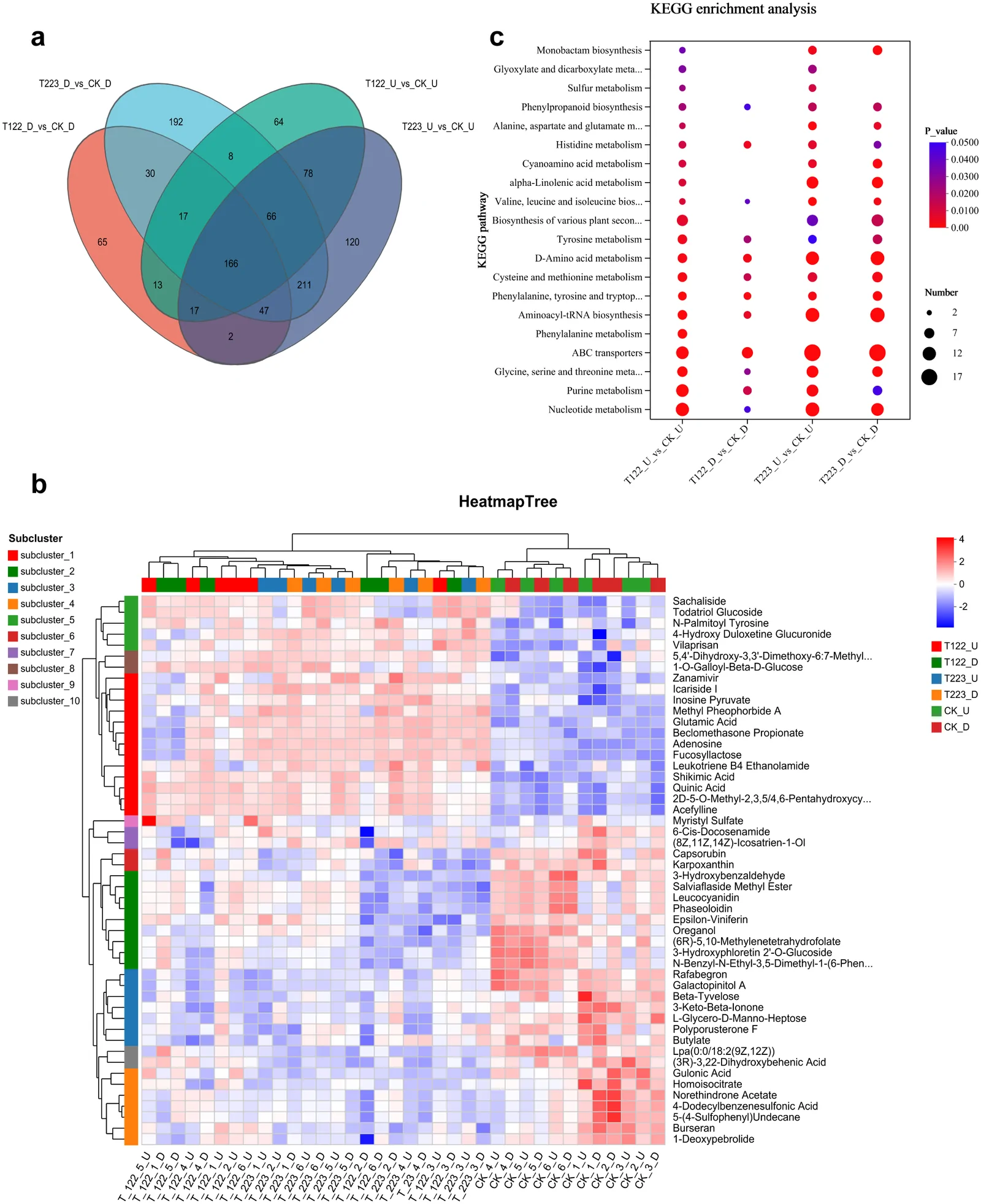
Effects of post-treatment with B122 and B223 on DMs during the disease resistance process of *P. thunbergii*. **(A)** Venn enrichment analysis of DMs. **(B)** Cluster analysis of DMs. **(C)** Top 20 Kyoto encyclopedia of KEGG enrichment analysis of multi-group DMs.

Both treatments were enriched for aromatic amino-acid and phenylpropanoid metabolism, providing cross-omics consistency with the transcriptomic results. B223 additionally showed enrichment of pyrimidine metabolism, alanine/aspartate/glutamate metabolism, cyanoamino-acid metabolism, and the biosynthesis of diverse plant secondary metabolites. These pathways are candidates associated with the stronger B223 disease-suppression phenotype; they do not demonstrate increased production or nematicidal activity of specific compounds.

## 4 Discussion

Pine wilt disease caused by *B. xylophilus* is one of the most devastating diseases affecting global forest ecosystems, making the development of environmentally friendly and sustainable control strategies an urgent priority (Cardoso et al., 2024; Du et al., 2024; Tahir et al., 2024). Beneficial rhizosphere microorganisms can contribute to disease management through multiple mechanisms, including host defense modulation, competition, niche exclusion, and secretion of bioactive metabolites (Arkhipov et al., 2024; Feng et al., 2021). Research has found that *B. subtilis JCK-1398* can inhibit the disease by inducing resistance in pine trees (Mannaa et al., 2024). *Bacillus* strains isolated from the rhizosphere of *Clerodendrum infortunatum* show potential in promoting plant growth and controlling plant diseases in field applications (Panichikkal et al., 2024). Furthermore, in pot experiments, *B. velezensis SDTB038* achieved a control efficacy of 42.98% against *Fusarium* crown and root rot in tomatoes, demonstrating the significant potential of discovering effective biocontrol resources from native environments (Chen et al., 2022). These cases collectively indicate that *Bacillus* species can contribute to plant protection, although the relative contribution of direct antagonism and host-mediated resistance may differ among strains and pathosystems. In this study, two *Bacillus* strains isolated from soil after logging disturbance *B. thuringiensis* B122 and *B. velezensis* B223 were systematically evaluated. Root-drenching treatment with these strains significantly reduced PWD symptoms in *P. thunbergii* following *B. xylophilus* inoculation, with B223 showing particularly high relative control efficacy (76.1%). Through integrated physiological, transcriptomic, and metabolomic analyses, we identified candidate molecular and metabolic responses associated with the differential performance of these two strains, especially B223.

Previous studies have indicated that forest logging disturbance can significantly alter soil microbial communities and nitrogen cycling (Li, 2024). The successful isolation of B122 and B223 in this research, along with their outstanding plant-growth-promoting and disease-resistant potential, may reflect the niche selection following such disturbance. Our disease phenotype evaluation demonstrated that root-drenching treatment delayed wilting progression after *B. xylophilus* infection and significantly reduced the disease index. This result is consistent with reports on *Bacillus*-based biocontrol agents in other plant systems (Guo et al., 2024; Li et al., 2019), but the present experimental design does not distinguish host-mediated induced resistance from possible direct effects on the nematode. This caveat is particularly important because *B. velezensis* is known to produce diverse lipopeptides and polyketides, whereas *B. thuringiensis* may produce toxins or other metabolites with potential nematicidal activity (Shi and Sun, 2025). Therefore, the higher control efficacy of B223 may reflect one or more mechanisms, including stronger root colonization, host defense modulation, or direct nematode inhibition by secreted bacterial products. Future *in vitro* nematicidal assays using bacterial cells, culture filtrates, and purified metabolites, together with split-root or bacterial wash-off experiments, will be required to determine whether symptom suppression is primarily direct, host-mediated, or a combination of both.

The dynamic changes in the plant defense enzyme system serve as important indicators of its immune status (Dos Santos and Franco, 2023; Vaghela et al., 2022). Studies have shown that *B. subtilis* JK-14 can significantly increase the activity of three defense enzymes, including superoxide dismutase, in peaches, thereby enhancing their resistance to *Alternaria tenuis* and *Botrytis cinerea* (Zhang et al., 2019). This study revealed that both treatments led to an increase in malondialdehyde (MDA) content, which typically indicates membrane lipid peroxidation—a common response under biotic stress (Morales and Munné-Bosch, 2024).The two strains also differed in hydrogen peroxide (H_2_O_2_) dynamics: B122 treatment resulted in sustained H_2_O_2_ accumulation, whereas B223 treatment maintained H_2_O_2_ near or slightly below the control level. H_2_O_2_ plays a dual role in plant defense. At moderate and spatially restricted levels, it functions as a signaling molecule that participates in defense gene expression, hormone signaling, and systemic response propagation; at excessive or prolonged levels, it may contribute to oxidative damage, membrane lipid peroxidation, and cellular dysfunction (Liao et al., 2025). Because this study did not measure cell death, electrolyte leakage, protein carbonylation, or other direct markers of oxidative injury, we cannot define a quantitative threshold separating beneficial defense signaling from damaging oxidative stress. Thus, the stronger H_2_O_2_ accumulation observed under B122 treatment should be interpreted as a distinct redox response rather than definitive evidence of a detrimental response. Similarly, the relatively stable H_2_O_2_ pattern under B223 treatment is consistent with ROS homeostasis but does not alone prove that this state is causally responsible for higher disease suppression. Recent studies on ROS/RNS biosynthesis and signal transduction have emphasized that effective pathogen defense requires precise regulation of ROS gradients, antioxidant capacity, and redox-dependent signaling (Muhammad Ali, 2025). Future work combining H_2_O_2_ imaging, oxidative damage assays, antioxidant enzyme profiling, and disease phenotyping will be needed to clarify how these redox patterns contribute to PWD suppression.

Transcriptomic analysis provided molecular-level associations that may help explain the physiological differences and the superior disease-suppressive phenotype of B223. Enrichment analysis of differentially expressed genes (DEGs) revealed that both B122 and B223 treatments were associated with multiple core pathways related to plant defense. Among these, the enrichment of the MAPK signaling pathway (map04016) was particularly critical, this pathway serves as a central hub for signal transduction and the initiation of defense gene expression upon plant perception of MAMPs (Wu et al., 2021). However, enrichment of MAPK-related annotations should be interpreted as evidence of pathway-level association rather than direct proof of pathway activation. The co-enrichment of phenylpropanoid biosynthesis (map00940) and flavonoid biosynthesis (map00941) is consistent with a role for secondary metabolism in the treatment response. The phenylpropanoid pathway provides precursors for lignin, flavonoids, and phenolic compounds (Lan et al., 2023; Wang et al., 2023), which may contribute to cell-wall reinforcement and antimicrobial metabolite production (Olivares-García et al., 2020). Strain-specific enriched pathways also suggested divergent host response patterns. B122 treatment specifically enriched the plant-pathogen interaction pathway (map04626) and glutathione metabolism pathway (map00480), the latter being consistent with the higher H_2_O_2_ levels observed under B122 treatment (Pi Lewska-Bednarek et al., 2018). In contrast, B223 treatment showed distinct enrichment of the plant hormone signal transduction pathway (map04075), which contains components annotated to jasmonic acid (JA), ethylene (ET), and salicylic acid (SA) signaling. Because JA/ET concentrations, canonical marker genes, and pathway perturbation assays were not measured in the present study, these data do not by themselves demonstrate JA/ET activation. Rather, they suggest that B223 treatment is associated with transcriptional changes in hormone-related pathways that merit targeted validation. This interpretation is consistent with previous studies showing that beneficial Bacillus strains can be associated with SA-, JA-, or ET-related defense responses in other hosts (Yu et al., 2024; Rosier et al., 2023; Mo et al., 2025). Needle physiology and RNA sequencing provided tissue-specific information on early, distal responses associated with root treatment. Such sampling is consistent with previous evidence that pine needles can exhibit systemic metabolic, hormonal, antioxidant, and defense-gene responses following microbial treatment or localized pathogen challenge (Hu et al., 2021; Mannaa et al., 2024; Wang et al., 2022c). However, systemic defense in pines can be organ dependent (Blodgett et al., 2007), and PWD pathology develops principally through nematode migration in stem resin canals and progressive xylem dysfunction (Yazaki et al., 2018; Umebayashi et al., 2011).

Metabolomic analysis provided complementary evidence for treatment-associated metabolic reprogramming. Previous studies have shown that *B. subtilis* TCX1 achieves an inhibition rate of up to 86.0% against cucumber *Fusarium* wilt pathogens by synthesizing and secreting various antimicrobial metabolites (Dong et al., 2025). A core *Bacillus* community with no direct lethal effect against *Fusarium* spp. in maize can induce the host to synthesize antimicrobial metabolites like berberine to resist stalk rot, revealing a novel pathway of indirect regulation of host metabolic defenses (Adeniji and Babalola, 2022). In our study, inoculation with pinewood nematodes induced significant metabolic reprogramming in the stems of *P. thunbergii* following treatments with either strain B122 or B223. The co-enriched pathways, including Phenylpropanoid Biosynthesis (map00940) and Phenylalanine, Tyrosine and Tryptophan Biosynthesis (map00400), were consistent with the transcriptomic enrichment patterns. Phenylalanine serves as the initial substrate for the phenylpropanoid pathway; the reinforcement of its biosynthetic pathway may support the synthesis of defense-related metabolites (Urban and Hura, 2023). The significant alterations in lipids and lipid-like molecules, primary components of membranes, are likely associated with defense signal transduction and membrane structural remodeling (Yu et al., 2021). Notably, treatment with strain B223 showed broader enrichment in pathways such as Pyrimidine Metabolism (map00240), Alanine, Aspartate and Glutamate Metabolism (map00250), and Biosynthesis of Various Plant Secondary Metabolites (map00999) revealed a more active and diverse metabolic state. Pyrimidine nucleotides are essential for RNA and DNA synthesis, and their active metabolism may support broader transcriptional and biosynthetic activity (Stasolla et al., 2003). The significant enrichment of the “Biosynthesis of Various Plant Secondary Metabolites” pathway suggests, but does not directly prove, increased synthesis of antimicrobial compounds such as phytoalexins and alkaloids. Targeted metabolite quantification and bioactivity assays will be needed to determine whether these metabolites directly suppress pinewood nematode activity or primarily reflect host stress responses.

It is also important to consider that the two strains compared in this study, B122 and B223, belong to different *Bacillus* species *B. thuringiensis* and *B. velezensis*, respectively. These species possess fundamentally distinct genomic repertoires that may intrinsically bias their plant-interaction phenotypes. *B. thuringiensis* is best known for its insecticidal Cry toxins and has been less frequently documented as a potent inducer of systemic resistance in woody plants, although certain strains can produce other secondary metabolites. In contrast, *B. velezensis* has emerged as a prominent plant-beneficial species with well-characterized genomic islands dedicated to the synthesis of diverse lipopeptides (surfactin, fengycin, iturin), polyketides (bacillaene, difficidin), and siderophores, many of which function as MAMPs or directly modulate plant hormone signaling (Yu et al., 2024; Ye et al., 2023). Some of these compounds may function as MAMP-like elicitors, plant signaling modulators, or direct antimicrobial/nematicidal factors (Zhang et al., 2022; Chen et al., 2022). Therefore, the superior control efficacy of B223 (76.1% vs. 40.8% for B122) and its unique activation of the plant hormone signal transduction pathway (map04075) may partially reflect species-level traits rather than purely strain-level variations. Conversely, the stronger H_2_O_2_-associated response observed under B122 treatment should not be interpreted as intrinsically detrimental without additional oxidative damage measurements. Future studies including multiple strains per species, comparative genomics, metabolite profiling, nematicidal assays, colonization assays, and mutant complementation will be required to dissect the relative contributions of species-level background, strain-specific adaptations, and host-mediated responses. Nonetheless, our results support *B. velezensis* B223 as a promising candidate for further development and testing as a biocontrol agent against PWD.

Integrating the multi-omics data, we propose a preliminary and testable working model for B223-associated suppression of pine wilt disease symptoms in *P. thunbergii*. After root application, B223 may colonize the rhizosphere and interact with pine roots through MAMPs, secreted metabolites, or both. These interactions may be associated with enrichment of hormone-related transcriptional pathways, including JA/ET-related annotations, together with MAPK signaling and phenylpropanoid/flavonoid metabolism. Such changes could contribute to transcriptional reprogramming, accumulation of lignin precursors, flavonoids, phytoalexin-like compounds, and other secondary metabolites, and possible reinforcement of physical or chemical barriers in stem tissues. However, because direct nematicidal activity, bacterial colonization, hormone levels, marker-gene expression, lignin deposition, and metabolite bioactivity were not directly tested, this model should be viewed as a hypothesis rather than a demonstrated causal mechanism. B122 may partially share these response patterns but was associated with stronger H_2_O_2_ accumulation and less extensive hormone-related/metabolic pathway enrichment in the present dataset. From an application perspective, B223 has several characteristics that support its potential development as a microbial biocontrol agent, including its affiliation with the spore-forming genus *Bacillus* and its disease-suppressive activity in seedling assays. Successful field deployment will require additional evaluation of spore production efficiency, formulation stability, shelf life, rhizosphere and endosphere colonization ability, compatibility with existing silvicultural practices, and performance under variable soil moisture, temperature, and nutrient conditions. Field trials should also assess whether root drenching, trunk injection, seedling substrate treatment, or combined application with other biological or chemical measures provides the most reliable control efficacy against PWD.

Based on our integrated multi-omics analysis, B122 and B223 were associated with distinct physiological, transcriptional, and metabolic response patterns in *P. thunbergii*, with B223 showing stronger disease-suppressive performance under greenhouse conditions. Our correlative data suggest that B223 treatment is associated with broader hormone-related transcriptional enrichment and secondary-metabolite reprogramming than B122 treatment. However, this study has several limitations. First, direct nematicidal or nematostatic activity of B122 and B223 was not tested, and therefore the observed disease suppression cannot be attributed exclusively to induced host resistance. Second, JA/ET pathway activation was inferred from KEGG enrichment only; JA and ET levels, canonical marker-gene expression, and functional pathway perturbation were not assessed. Third, although H_2_O_2_ dynamics differed between strains, no quantitative threshold or independent oxidative damage markers were measured to classify the response as beneficial signaling versus damaging oxidative stress. Fourth, RNA-seq-based DEG patterns were not independently validated by qRT-PCR, targeted metabolite quantification, or functional genetic approaches. Fifth, only one *B. xylophilus* isolate, Nxy61, was used, which limits the generalizability of the results across nematode populations. Sixth, the experiments were conducted under controlled greenhouse conditions, which may not fully reflect complex field environments. Future studies should address these limitations using *in vitro* nematicidal assays, split-root and bacterial wash-off experiments, larger sample sizes, multiple nematode isolates, time-course sampling, qRT-PCR validation, hormone quantification, targeted metabolite quantification, bacterial genome analysis, colonization assays, oxidative damage assays, and field trials under different environmental conditions. These efforts will help clarify the causal mechanisms underlying Bacillus-associated PWD suppression and support the development of reliable microbial formulations.

## Data Availability

The datasets presented in this study can be found in online repositories. The names of the repository/repositories and accession number(s) can be found below: https://www.ncbi.nlm.nih.gov/, PRJNA1429861 https://www.ncbi.nlm.nih.gov/, PRJNA1429863.

## Supplementary material

Supplementary Figure 1 Screening of biocontrol bacteria. **(A)** Nitrogen-fixing capacity assay. **(B)** Phosphorus-solubilizing ability assay.

Supplementary Figure 2 Screening of biocontrol bacteria. **(A)** Hemolytic activity assay. **(B)** Indole-3-acetic acid (IAA) production capacity assay. **(C)** Motility assay.

Supplementary Figure 3 Transcriptomic comparative analysis of strains B122 and B223. **(A)** Counts of expressed genes between two groups. **(B)** Venn analysis of DEGs between two groups across time points. **(C)** GO enrichment analysis. **(D)** KEGG enrichment analysis.

Supplementary Figure 4 RT-qPCR validation of mRNA-seq sequencing data.

Supplementary Figure 5 Metabolic effects of post-treatment with B122 and B223 during the disease resistance process of *P. thunbergii*. **(A)** Principal Component Analysis. **(B)** Exploratory PLS-DA score plot; model-quality metrics were R2X(cum) = 0.262, R2Y(cum) = 0.268, and Q2(cum) = 0.161. **(C)** Bar chart of counts of DMs. **(D)** Pie chart of metabolite categories.

Supplementary Table 1 mRNA Quantitative Primers.

Supplementary Table 2 Bacterial isolation and identification.

Supplementary Table 3 Statistical analyses and mapping results of RNA sequencing reads.
