# Supplementary material for: Multi-omics analysis of Bacillus-associated suppression of Bursaphelenchus xylophilus disease symptoms and candidate defense responses in Pinus thunbergii
Source: Front Plant Sci. 2026 Oct 14;17:1854108. doi: 10.3389/fpls.2026.1854108 (PMC13715560; doi:10.3389/fpls.2026.1854108)
Supplement: Supplementary file 1 [file DataSheet1.docx]

**Table Legends：**

Table.S1 mRNA Quantitative Primers

| **Name** | **Sequence** |
| --- | --- |
| qCHS-F | AGATCACCCATGTCGTTTTC |
| qCHS-R | CCCTGTTGGTACATCATGAC |
| qC4H-F | CCAACCCACTTCTTTACTGG |
| qC4H-R | TGTTCACACCATCGGTAATG |
| qGSTU-F | GAAGTGATGCCTGATTTTGC |
| qGSTU-R | TGGTTTGCCCTATTTAGCTG |
| qbHLH46-F | AGATGGATAAGGCCTCTCTG |
| qbHLH46-R | TTCTTCAAGGCCTCAAGTTG |
| qNRT2-F | TTCACCTCTCTTGGATCTCC |
| qNRT2-R | GTTGCCTATGTCCTTCTGTG |
| qACS-F | TAAAGGCAGCACAACAGATG |
| qACS-R | AATGTCCAGCTTTTCCATGG |
| qERECTA-F | CGAGATTCGTTTCCAGACTC |
| qERECTA-R | TTTATCTACCACGCATGCTG |
| qCHI1-F | AATGTCGGGGTTCTTTATGC |
| qCHI1-R | TCTGGAAAGAGCTACTACGG |
| qERF1-like-F | GCCTCTGCTCCTTTCATAAC |
| qERF1-like-R | TCACGCCAAAATATCGTCAG |
| qU2af-F | AGAATACAAGGTCCGGAGAG |
| qU2af-R | CAAAAGTGCTTGCTGTTCAG |

Table.S2 Bacterial isolation and identification

| Sample plot | Species name | Bacterial species | Bacterial population |
| --- | --- | --- | --- |
| Sample 1 | *Pinus massoniana* | *Bacillus velezensis* | 9 |
|  |  | *Bacillus amyloliquefaciens* | 4 |
|  | *Gardenia jasminoides* | *Bacillus velezensis* | 8 |
|  |  | *Bacillus thuringiensis* | 7 |
|  | *Symplocos tanakana* | *Bacillus velezensis* | 11 |
|  | *Liriodendron chinense* | *Bacillus velezensis* | 8 |
|  | *Cunninghamia lanceolata* | *Bacillus velezensis* | 7 |
|  |  | *Bacillus amyloliquefaciens* | 6 |
|  | *Quercus variabilis Blume* | *Bacillus amyloliquefaciens* | 12 |
| Sample 2 | *Cunninghamia lanceolata* | *Bacillus subtilis* | 8 |
|  | *Pinus sylvestris var.mongholica* | *Bacillus amyloliquefaciens* | 7 |
|  |  | *Bacillus velezensis* | 11 |
|  | *Sassafras tzumu* | *Bacillus velezensis* | 15 |
|  | *Liriodendron chinense* | *Bacillus subtilis* | 6 |
|  | *Litsea cubeba* | *Bacillus velezensis* | 13 |
|  | *Pinus massoniana* | *Bacillus velezensis* | 8 |
| Sample 3 | *Dalbergia hupeana* | *Bacillus amyloliquefaciens* | 17 |
|  | *Eurya loquaiana* | *Bacillus subtilis* | 9 |
|  |  | *Bacillus amyloliquefaciens* | 13 |
|  | *Vernicia fordii* | *Bacillus velezensis* | 15 |
|  | *Albizia kalkora* | *Bacillus subtilis* | 9 |
|  | *Myrsine africana* | *Bacillus velezensis* | 11 |

Table.S3 Statistical analyses and mapping results of RNA sequencing reads

| Sample | Time points after infection | Rep | Raw reads | Raw bases | Clean reads | Clean bases | Error rate(%) | Q20(%) | Q30(%) | GC content(%) |
| --- | --- | --- | --- | --- | --- | --- | --- | --- | --- | --- |
| CK | 1d | 1 | 40079898 | 6052064598 | 39847522 | 5988743392 | 0.0119 | 98.86 | 96.23 | 45.16 |
|  |  | 2 | 42009362 | 6343413662 | 41750594 | 6278498859 | 0.0118 | 98.92 | 96.43 | 45.08 |
|  |  | 3 | 46632746 | 7041544646 | 46377660 | 6971122962 | 0.0118 | 98.92 | 96.42 | 44.99 |
| B122 | 1d | 1 | 41116010 | 6208517510 | 40899974 | 6151228301 | 0.0118 | 98.88 | 96.27 | 45.02 |
|  |  | 2 | 42604176 | 6433230576 | 42360034 | 6373463789 | 0.0119 | 98.87 | 96.24 | 45.21 |
|  |  | 3 | 48180256 | 7275218656 | 47883332 | 7198456070 | 0.0118 | 98.9 | 96.36 | 45.18 |
|  | 3d | 1 | 48906864 | 7384936464 | 48612256 | 7309808119 | 0.0118 | 98.9 | 96.36 | 45.27 |
|  |  | 2 | 47202920 | 7127640920 | 46924464 | 7054893157 | 0.0118 | 98.92 | 96.43 | 45.28 |
|  |  | 3 | 43350678 | 6545952378 | 43090470 | 6478661260 | 0.0118 | 98.9 | 96.35 | 45.28 |
|  | 5d | 1 | 41903220 | 6327386220 | 41723920 | 6277095122 | 0.0117 | 98.98 | 96.57 | 45.34 |
|  |  | 2 | 45162966 | 6819607866 | 44901150 | 6755870113 | 0.0117 | 98.93 | 96.44 | 45.31 |
|  |  | 3 | 49065476 | 7408886876 | 48810714 | 7339651730 | 0.0118 | 98.92 | 96.4 | 45.23 |
|  | 7d | 1 | 47240498 | 7133315198 | 46939586 | 7057388609 | 0.0118 | 98.87 | 96.25 | 45.36 |
|  |  | 2 | 53051380 | 8010758380 | 52752446 | 7934417060 | 0.0118 | 98.9 | 96.36 | 45.22 |
|  |  | 3 | 43299778 | 6538266478 | 43054684 | 6477407916 | 0.0118 | 98.88 | 96.28 | 45.29 |
| B223 | 1d | 1 | 49548608 | 7481839808 | 49255446 | 7407856277 | 0.0118 | 98.9 | 96.33 | 45.11 |
|  |  | 2 | 50071428 | 7560785628 | 49785512 | 7491560701 | 0.0118 | 98.88 | 96.29 | 45.16 |
|  |  | 3 | 48920736 | 7387031136 | 48617212 | 7309026228 | 0.0118 | 98.9 | 96.35 | 45.16 |
|  | 3d | 1 | 48754334 | 7361904434 | 48452670 | 7284141168 | 0.0118 | 98.9 | 96.36 | 45.22 |
|  |  | 2 | 52289526 | 7895718426 | 51983646 | 7816218678 | 0.0118 | 98.9 | 96.37 | 45.32 |
|  |  | 3 | 48435582 | 7313772882 | 48163216 | 7235941509 | 0.0118 | 98.89 | 96.33 | 45.32 |
|  | 5d | 1 | 45891482 | 6929613782 | 45632380 | 6863355526 | 0.0118 | 98.89 | 96.33 | 45.33 |
|  |  | 2 | 45726326 | 6904675226 | 45464230 | 6838507130 | 0.0118 | 98.89 | 96.31 | 45.17 |
|  |  | 3 | 45880152 | 6927902952 | 45632406 | 6862624684 | 0.0118 | 98.89 | 96.31 | 45.07 |
|  | 7d | 1 | 54441686 | 8220694586 | 54129110 | 8144607246 | 0.0118 | 98.92 | 96.42 | 45.29 |
|  |  | 2 | 49497682 | 7474149982 | 49228370 | 7402833351 | 0.0118 | 98.91 | 96.39 | 45.18 |
|  |  | 3 | 51885490 | 7834708990 | 51591606 | 7756833546 | 0.0117 | 98.93 | 96.46 | 45.15 |
